# Clinical predictors of physiologic change after treatment with immunosuppression in hypersensitivity pneumonitis

**DOI:** 10.1371/journal.pone.0313540

**Published:** 2024-11-25

**Authors:** Margaret Kypreos, Esther de Boer, Graham Ellington, Genichiro Fujioka, Jerry Liu, Craig Glazer, Traci Adams

**Affiliations:** 1 Division of Pulmonary and Critical Care Medicine, University of Texas Southwestern Medical Center, Dallas, Texas, United States of America; 2 Critical Care and Pulmonary Consultants P.C., Greenwood Village, Colorado, United States of America; 3 Department of Medical Education, Texas A&M University School of Medicine, Bryan, Texas, United States of America; SMS Medical College and Hospital, INDIA

## Abstract

**Introduction:**

Treatment of hypersensitivity pneumonitis involves removal of the antigen and may include the use of immunosuppression or antifibrotic therapy. It remains unclear whether antifibrotic or immunosuppressive therapy is more beneficial in fibrotic hypersensitivity pneumonitis or if clinical markers can predict a patient’s response to therapy.

**Methods:**

We evaluated a retrospective cohort in order to determine if certain clinical characteristics can predict physiologic improvement with immunosuppressive treatment in patients with chronic hypersensitivity pneumonitis. Patients with a diagnosis of hypersensitivity pneumonitis with a moderate, high, or definite confidence according to the American Thoracic Society criteria were included in the study.

**Results:**

Overall immunosuppression did not lead to improvement in % predicted forced vital capacity (FVC%) and % predicted diffusion capacity (DLCO%). Patients with fibrotic hypersensitivity pneumonitis and those with familial interstitial lung disease demonstrated a decline in FVC% predicted as well as DLCO% predicted over one year and the use of immunosuppression does not modify that risk. In contrast, patients with extensive ground glass demonstrated improvement in DLCO% predicted but not FVC% predicted over one year with or without the use of immunosuppression.

**Conclusion:**

Our study demonstrates that certain radiographic variables trend toward a significant impact on FVC% predicted as well as DLCO% predicted and suggests that antifibrotic therapy may be a better initial choice of therapy in patients with fibrotic hypersensitivity pneumonitis as decline occurred with or without the use of immunosuppression.

## Introduction

Hypersensitivity pneumonitis (HP), or extrinsic allergic alveolitis, is an immunologically mediated interstitial lung disease (ILD) that involves terminal bronchioles, interstitium, and alveoli [1]. HP results from repeated exposures to an aerosolized antigen [2]. Genetic and environmental risk factors increase susceptibility for HP [3].

Treatment involves removal of the antigen which has been shown to improve survival [4, 5]. Immunosuppression has historically been the first-line treatment option for patients with HP. The use of short-term immunosuppression first occurred in farmer’s lung and it was shown that diffusion capacity improved after one month of treatment [6]. Longer-term studies of immunosuppression have demonstrated a slowing in the decline of lung function in patients on mycophenolate or azathioprine [7].

Antifibrotic therapy has been approved for the treatment of HP in patients with a progressive fibrotic phenotype based on the INBUILD trial; however, because immunosuppression was not permitted at enrollment in INBUILD, it remains unclear whether antifibrotic or immunosuppressive therapy is more beneficial in fibrotic HP or whether any clinical markers can predict a patient’s response to therapy [8]. Data is needed to guide selection of therapy for patients with HP in order to maximize treatment response while minimizing adverse events.

The objective of this retrospective study is to determine whether clinical characteristics, including demographics, radiographic findings, or histopathology can predict response to immunosuppressive treatment in chronic HP.

## Methods

We retrospectively identified ILD patients evaluated from 2003–2018 from the University of Texas Southwestern Medical Center (UTSW). This study was conducted in accordance with the amended Declaration of Helsinki, and the UTSW Institutional Review Board approved the study (STU-2019-0913).

A registry in Epic which included all patients seen in the UTSW Interstitial Lung Disease Clinic was created. For the purposes of this study, the diagnosis of all patients from the Epic registry were re-confirmed by review of the medical record, imaging, and histopathology in a multidisciplinary discussion. Patients with a diagnosis of HP with a moderate, high, or definite confidence according to the American Thoracic Society (ATS) criteria were included in the study [9]. Patients with non-HP diagnoses were excluded.

Demographics such as age, gender, race, ethnicity, and smoking status were extracted from the medical record. Providers used a detailed questionnaire to ascertain exposure. Avian antigen was documented if there was consistent exposure to a live bird or feather products. Mold antigen was documented if the patient was persistently exposed to visible mold either at home or in the office or consistently used composte heap. For all exposures, a temporal relationship was established, and exposures were only deemed significant if they were persistent and preceded the development of ILD. The exposure history was reviewed by an occupational medicine specialist in cases where it was unclear if the exposure was significant enough to lead to sensitization. Bronchoscopy results, including lymphocyte percentage from bronchoalveolar lavage and histopathology from transbronchial biopsy, were extracted from the medical record. Bronchoalveolar lavage (BAL) lymphocytosis was defined as greater than 30% lymphocytes. Pathological findings of granulomas, giant cells, interstitial inflammation, and airway centric findings were recorded. Completion of a surgical lung biopsy with the final pathologist diagnosis were extracted. The presence of granulomas, giant cells, interstitial inflammation, and airway centric findings were also recorded. High resolution computed tomography (HRCT) images were reviewed by a thoracic radiologist who was blinded to the clinical diagnosis. The severity of fibrosis was stratified into mild, moderate, and severe based on the degree of fibrotic involvement. Fibrosis was severe if > 50% of the parenchyma was fibrotic, moderate if 10–50% was fibrotic, and mild if < 10% was fibrotic [10]. Findings of reticulation, traction bronchiectasis, honeycombing, mosaicism, air trapping, and extensive ground glass were recorded. The GAP score was calculated for each patient. A family history of interstitial lung disease was noted as well as if serologic criteria for interstitial pneumonia with autoimmune features was met. Immunosuppression regimens included mycophenolate mofetil, azathioprine, and prednisone. Date of initiation and completion were documented. Pulmonary function testing data included the absolute as well as % predicted forced vital capacity (FVC%) and diffusion capacity (DLCO%).

Paired T test of the entire cohort evaluating the change in percent predicted FVC and DLCO was completed. Univariable linear regression was then completed with variables that included demographic, radiographic, serologic, and pathological findings on transbronchial and surgical lung biopsies. Variables with p < 0.1 in the univariable model were included in a multivariable linear regression model. Only variables with p < 0.05 were considered significant.

Additional analysis was then completed only on patients initiated on immunosuppression within the first year of establishing care in the pulmonary clinic at UTSW. None of these patients were on immunosuppression prior to initiation during a clinic appointment at UTSW. Univariable linear regression was completed with variables that included demographic, radiographic, serologic, and pathological findings on transbronchial and surgical lung biopsies. Variables with p < 0.1 in the univariable model were included in a multivariable linear regression model. Only variables with p < 0.05 were considered significant.

## Results

Our ILD cohort from the Epic registry contains 1100 patients. Of these, 264 patients had a moderate, high, or definite confidence diagnosis of HP by ATS criteria. Of those with HP, 50.3% were male and 80.6% were white. Antigen was identified in 86.7% of patients with avian being the most common in 56.1% of cases. Baseline average FVC% predicted was 67.4% and average DLCO% predicted was 48.5%. Reticulations and traction bronchiectasis were evident on the high resolution CT chest in 84.1% and 78.4% of patients respectively. A lung biopsy that was either transbronchial or surgical was completed in 91.3% of cases (Table 1).

**Table 1:** Characteristics of entire cohort.

Mean Age (SD)	64
Male, No. (%)	133 (50.3%)
Ethnicity, No. (%)	
White	213 (80.6%)
Black	4 (1.5%)
Hispanic or Latino	16 (6.1%)
Asian	8 (3.0%)
Other	1 (0.5%)
Unknown	10 (3.8%)
Ever Smoker, No. (%)	126 (47.7%)
Pack years, median (IQR)	24 (25.75)
Antigen Identified, No. (%)	229 (86.7%)
Avian/Feather	148 (56.1%)
Mold	111 (42.0%)
Other ***	28 (10.6%)
Baseline Lung Function, mean	
FVC % predicted (SD)	67.4% (22)
DLCO% predicted (SD)	48.5% (22)
HRCT Fibrosis Severity	
Mild Fibrosis	103 (39.0%)
Moderate Fibrosis	87 (33.0%)
Severe Fibrosis	37 (14.0%)
HRCT Reticulations	222 (84.1%)
HRCT Traction Bronchiectasis	207 (78.4%)
HRCT Honeycombing	74 (28.0%)
HRCT Extensive Ground Glass	137 (52.0%)
HRCT Profuse Micronodules	42 (15.9%)
HRCT Air Trapping	153 (58.0%)
Bronchoalveolar Lavage	87 (33.0%)
Lung Biopsy Performed, No. (%)	241 (91.3%)
Transbronchial Biopsy	101 (38.3%)
Surgical Lung Biopsy	140 (53.0%)
Clinical Outcomes	
Death	45 (17.0%)
Average Time to Death (Years)	
Transplant	35 (13.3%)
Average Time to Transplant (Years)	

***Other antigens include isocyanate exposure and fish tank exposure

There was no significant difference between FVC % predicted or DLCO % predicted prior to treatment compared to after 1 year of treatment (p = 0.88 and p = 0.56, respectively). P-interaction between immunosuppression and FVC % predicted and between immunosuppression and DLCO % predicted was non-significant.

Univariable analysis of change in FVC% predicted at baseline and at 12 months in the entire cohort showed a significant decline in FVC% in patients with irregular reticulations on HRCT (p<0.001), familial ILD (p = 0.006), and MMF use (0.006) (Table 2). A significant improvement in DLCO% predicted in patients with extensive ground glass opacities (p = 0.01) and air trapping (p = 0.008), while a significant decline in DLCO % predicted was seen in patients with irregular reticulations on HRCT (p<0.001), higher GAP score (p = 0.02), and MMF use (p = 0.005) (Table 3). In the multivariable model, irregular reticulations were included but not traction bronchiectasis due to collinearity. Multivariable analysis of change in FVC% predicted showed a significant decline in patients with reticulation (p = 0.04), a family history of interstitial lung disease (p = 0.003), and those on mycophenolate (p = 0.0002). A significant decline in DLCO% predicted was also seen in patients with reticulation (p = 0.0002) and MMF use (p = 0.017), and a significant improvement was seen in patients with extensive ground glass opacities (p = 0.01).

**Table 2:** Univariable and multivariable analysis of change in FVC % predicted at baseline and after 12 months of entire cohort.

Variable	Univariable analysis			Multivariable analysis		
	Estimate	95% CI	P value	Estimate	95% CI	P value
Age at diagnosis	-0.03	-0.14 to 0.07	0.54			
Male Gender	-0.98	-3.42 to 1.47	0.43			
White Race	2.54	-0.89 to 5.97	0.15			
Smoking status at diagnosis	-0.51	-2.96 to 1.95	0.68			
Antigen identified	2.61	-1.06 to 6.27	0.16			
BAL lymph > 30%	0.1151	-0.017 to 0.25	0.087			
TBBx with granulomas	1.80	-2.62 to 6.21	0.42			
HRCT fibrosis severity						
Nonfibrotic	[ref]					
Mild fibrosis	-6.41	-10.11 to -2.70	0.008			
Moderate fibrosis	-8.30	-12.1 to -4.5	<0.001			
Severe fibrosis	-5.83	-10.36 to -1.29	0.01			
HRCT reticulations	-6.93	-10.18 to -3.68	<0.001	-0.14	-0.27 to -0.008	0.04
HRCT traction bronchiectasis	-5.93	-8.82 to -3.05	<0.001			
HRCT honeycombing	-1.46	-4.18 to 1.26	0.29			
HRCT Extensive ground glass	2.14	-0.30 to 4.58	0.09			
HRCT Profuse micronodules	2.87	-0.44 to 6.17	0.09			
HRCT Air trapping	1.79	-0.71 to 4.29	0.16			
Familial ILD	-5.44	-9.33 to -1.56	0.006	-0.22	-0.37 to -0.075	0.0033
Positive CTD serologies	1.48	-1.34 to 4.29	0.30			
Gap Score	-0.26	-1.08 to 0.56	0.54			
MMF	-4.35	-6.83 to -1.88	0.006	-0.19	-0.29 to -0.093	0.0002
AZA	-1.15	-4.58 to 2.27	0.51			
Prednisone	-0.77	-3.38 to 1.84	0.56			

**Table 3:** Univariable and multivariable analysis of change in DLCO % predicted at baseline and after 12 months of entire cohort.

Variable	Univariable analysis			Multivariable analysis		
	Estimate	95% CI	P value	Estimate	95% CI	P value
Age at diagnosis	-0.01	-0.18 to 0.15	0.823			
Male gender	-2.53	-6.33 to 1.27	0.19			
White Race	2.13	-3.18 to 7.44	0.43			
Smoking status at diagnosis	-1.74	-5.55 to 2.07	0.37			
Antigen identified	3.00	-2.75 to 8.68	0.31			
BAL lymph > 20%	2.17	-2.801to 7.14	0.39			
BAL lymph > 30%	2.41	-2.90 to 7.72	0.37			
TBBx with granulomas	4.86	-1.18 to 10.90	0.11			
HRCT fibrosis severity						
Mild.	-11.21	-16.88 to -5.54	0.0001			
Moderate	-15.33	-21.13 to -9.53	<0.001			
Severe	-14.40	-21.34 to -7.47	<0.001			
HRCT reticulations	-14.37	-19.29 to -9.45	<0.001	-2.63	-3.99 to -1.27	0.0002
HRCT traction bronchiectasis	-12.54	-16.91to -8.16	<0.001			
HRCT honeycombing	-3.79	-8.01 to 0.43	0.08			
Extensive ground glass	4.81	1.03 to 8.59	0.01	1.24	0.30 to 2.18	0.0097
Profuse micronodules	3.32	-1.84 to 8.49	0.20			
Air trapping	5.15	1.34 to 8.95	0.008	0.24	-0.74 to 1.23	0.63
SLB granulomas	-0.65	-5.77 to 4.46	0.80			
Familial ILD	-2.03	-8.16 to 4.10	0.52			
Positive CTD serologies	0.08	-4.31 to 4.48	0.97			
GAP score	-1.60	-2.91 to -0.26	0.02	-0.023	-0.33 to 0.29	0.88
MMF	-1.586	-2.47 to -0.699	0.005	-1.18	-2.14 to -0.21	0.017
AZA	-0.95	-6.29 to 4.38	0.72			
Prednisone	-1.43	-5.49 to 2.64	0.49			

A total of 108 patients were initiated on immunosuppression within one year of the initial pulmonary clinic visit. Univariable analysis of the change in FVC% predicted at baseline and after twelve months of immunosuppression showed a significant decline in patients with mild and moderate degrees of fibrosis (p = 0.0129 and p = 0.0018), irregular reticulations on high resolution CT chest (p = 0.0042), and traction bronchiectasis on high resolution CT chest (p = 0.0065) (Table 4). A significant decline in DLCO% predicted was also seen in patients with any degree of fibrosis, irregular reticulations on high resolution CT chest (p = 0.0014), and traction bronchiectasis on high resolution CT chest (p = 0.0018) (Table 5). In the multivariable model, irregular reticulations were included but not traction bronchiectasis or degree of fibrosis due to collinearity. Multivariable linear regression showed a significant decline in FVC% predicted and DLCO% predicted in patients with irregular reticulations on high resolution CT chest (p = 0.04 and p = 0.003, respectively). Extensive ground glass was associated with improvement in DLCO % predicted in the multivariable model (p = 0.04).

**Table 4:** Univariable and multivariable analysis of change in FVC % predicted at baseline and after 12 months of immunosuppressive treatment of cohort on immunosuppression at one year.

Variable	Univariable analysis			Multivariable analysis		
	Estimate	95% CI	P value	Estimate	95% CI	P value
Male Gender	-2.19	-6.24 to 1.87	0.29			
White Race	2.78	-2.85 to 8.41	0.33			
Smoking status at diagnosis	-2.35	-6.42 to 1.72	0.26			
Antigen identified	2.30	-3.92 to 8.52	0.47			
BAL lymph > 20%	-0.90	-6.70 to 4.90	0.76			
BAL lymph > 30%	1.21	-5.27 to 7.68	0.71			
TBBx with granulomas	4.29	-3.87 to 12.45	0.29			
HRCT fibrosis severity						
Nonfibrotic	[ref]					
Mild fibrosis	-8.63	-15.39- -1.87	0.0129			
Moderate fibrosis	-10.84	-17.54 to -4.14	0.0018			
Severe fibrosis	-6.19	-13.60 to 1.21	0.10			
HRCT reticulations	-8.35	-14.01 to -2.69	0.0042	-0.24	-0.47 to -0.01	0.04
HRCT traction bronchiectasis	-7.38	-12.66 to -2.11	0.0065			
HRCT honeycombing	-0.58	-4.97 to 3.81	0.79			
HRCT Extensive ground glass	2.05	-2.03 to 6.14	0.32			
HRCT Air trapping	3.05	-1.9 to 7.18	0.15	0.04	-0.12 to 0.21	0.60
Familial ILD	-4.39	-11.05 to 2.27	0.19	-0.24	-0.49 to 0.006	0.06
Positive CTD serologies	0.66	-4.09 to 5.41	0.78			
Gap Score	0.54	-0.87 to 1.95	0.45			

**Table 5:** Univariable and multivariable analysis of change in DLCO % predicted at baseline and after 12 months of immunosuppressive treatment of cohort on immunosuppression at one year.

Variable	Univariable analysis			Multivariable analysis		
	Estimate	95% CI	P value	Estimate	95% CI	P value
Male Gender	-0.88	-6.83 to 5.07	0.77			
White Race	0.75	-7.32 to 8.82	0.85			
Smoking status at diagnosis	-3.21	-9.51 to 2.74	0.29			
Antigen identified	1.48	-7.62 to 10.58	0.75			
BAL lymph > 20%	-1.97	-10.53 to 6.59	0.65			
BAL lymph > 30%	-2.68	-12.23 to 6.87	0.58			
TBBx with granulomas	43.87	-6.09 to 12.83	0.44			
HRCT fibrosis severity						
Mild.	-13.20	-23.19 to -3.21	0.0101			
Moderate	-14.36	-24.25 to -4.46	0.0049			
Severe	-13.43	-24.38 to -2.49	0.0167			
HRCT reticulations	-13.55	-21.76 to -5.34	0.0014	-2.96	-4.90 to -1.03	0.003
HRCT traction bronchiectasis	-12.31	-19.94 to -4.68	0.0018			
HRCT honeycombing	-1.10	-7.52 to 5.32	0.08			
Extensive ground glass	4.64	-1.29 to 10.58	0.12	1.47	0.052 to 2.89	0.04
Air trapping	4.15	-1.77 to 10.06	0.17	-0.034	-1.50 to 1.43	0.96
Familial ILD	-2.62	-12.41 to 7.16	0.60			
Positive CTD serologies	-3.76	-10.65 to 3.13	0.28			
GAP score	-0.08	-2.14 to -1.99	0.94			

## Discussion

In this study, we aimed to determine whether clinical characteristics could predict physiologic improvement with immunosuppression in patients which chronic hypersensitivity pneumonitis. Immunosuppression did not lead to improvement in FVC % predicted and DLCO % predicted in patients with HP. Patients with fibrotic HP and those with familial ILD demonstrated decline in FVC and DLCO % predicted over 1 year, and the use of immunosuppression does not modify that risk. In contrast, patients with extensive ground glass demonstrated improvement in DLCO % predicted but not FVC % predicted over 1 year with or without the use of immunosuppression.

Our results fit with existing data on the use of immunosuppression in patients with HP and add to the literature by suggesting features of patients with HP who are likely to respond to immunosuppression. Studies have shown that corticosteroids alone do not lead to improvement in FVC or DLCO % predicted [7, 11]. In one study, addition of MMF or AZA led to stabilization of FVC predicted without overall improvement in PFTs at 1 year [12]. Our results provide further support for the previous findings as the majority of patients in our cohort were on either mycophenolate or azathioprine with or without the addition of prednisone.

Our study adds to the literature by specifying features of patients with HP that may modify response to immunosuppression. Patients with fibrotic HP declined with or without the use of immunosuppression. This data suggests that antifibrotic therapies, which have been demonstrated to slow the decline of patients with progressive fibrotic ILD [8, 13], may be a better initial choice of therapy than immunosuppression among patients with fibrotic HP. Our data also suggests that the majority of patients with fibrotic HP will demonstrate PFT decline at 1 year; however, antifibrotic therapy is approved only for the progressive fibrotic phenotype. We suggest that clinical trials to assess the efficacy of antifibrotic therapy in fibrotic HP at initial presentation are needed; by waiting to initiate antifibrotic therapy until patients demonstrate decline, we may miss a valuable treatment window.

Our data also suggests that patients with familial ILD are likely to decline with or without the use of immunosuppression. Our data fits with existing studies demonstrating higher mortality in familial ILD compared to non-familial ILD [14]. Our study did not demonstrate a worsening decline in lung function among the subset of familial ILD patients on immunosuppression. The clinical implication of this is that taking a thorough family history in HP is an essential part of the evaluation. Familial ILD is often associated with short leukocyte telomere length (LTL). Patients with short LTL demonstrate a faster decline in lung function than those with normal LTL; short LTL patients also have an increased mortality but not a change in lung function trajectory compared to patients with normal LTL [14, 15]. Our data fits with this observation and suggests that where LTL testing is unavailable, a family history may serve as a valuable clinical predictor of progression. PFT trajectory on immunosuppression cannot be used as a predictor of short LTL.

Finally, our study contributes to the literature by demonstrating that patients with extensive ground glass may improve with immunosuppression. While this is intuitive, it has not been previously demonstrated in patients with HP.

Strengths of our study include that patients had a well-defined diagnosis of HP in accordance with ATS guidelines. Our patients were not on concomitant antifibrotic therapy with nintendanib because nintedanib had not yet been approved for progressive fibrotic ILD at the time of our cohort, and therefore only the impact of immunosuppression was assessed. There are limitations of the study that should be acknowledged. This is a retrospective study thus compliance with immunosuppression could not be assessed. Additionally, not all of the patients were on immunosuppression which limited the overall cohort of patients on therapy. The importance of antigen removal was also discussed at the initial appointment, however it is difficult to determine if definitive removal of the antigen occurred given the retrospective nature of the study. Finally, the cohort is from a single quaternary referral center with expertise in ILD thus inclusion from other centers with ILD expertise is required for external validation.

## Conclusion

Our study showed that even though there is no overall impact of immunosuppression in the FVC% predicted or DLCO% predicted, certain radiographic variables trend toward a significant impact. Thus, patients with evidence of fibrosis on imaging may benefit from earlier initiation of antifibrotic therapy with nintendanib rather than immunosuppression.

## Supporting information

S1 Data Excel spreadsheet of De-identified data. (XLSX)
